# A Novel Metric “Exercise Cardiac Load” Proposed to Track and Predict the Deterioration of the Autonomic Nervous System in Division I Football Athletes

**DOI:** 10.3390/jfmk8040143

**Published:** 2023-10-07

**Authors:** S. Howard Wittels, Eric Renaghan, Michael Joseph Wishon, Harrison L. Wittels, Stephanie Chong, Eva Danielle Wittels, Stephanie Hendricks, Dustin Hecocks, Kyle Bellamy, Joe Girardi, Stephen Lee, Tri Vo, Samantha M. McDonald, Luis A. Feigenbaum

**Affiliations:** 1 Department of Anesthesiology, Mount Sinai Medical Center, Miami, FL 33140, USA; shwittels@gmail.com; 2 Department of Anesthesiology, Wertheim School of Medicine, Florida International University, Miami, FL 33199, USA; 3 Miami Beach Anesthesiology Associates, Miami, FL 33140, USA; 4 Tiger Tech Solutions, Inc., Miami, FL 33140, USA; joe@tigertech.solutions (M.J.W.); hl@tigertech.solutions (H.L.W.); schong591@gmail.com (S.C.); evadanielle@gmail.com (E.D.W.); steph.hendricks@gmail.com (S.H.); dustin@tigertech.solutions (D.H.); 5 Department of Athletics, Sports Science, University of Miami, Miami, FL 33146, USA; eric.renaghan@miami.edu (E.R.); lfeigenbaum@med.miami.edu (L.A.F.); 6 Department of Physical Therapy, Miller School of Medicine, University of Miami, Miami, FL 33146, USA; k.bellamy1@umiami.edu (K.B.); j.girardi@miami.edu (J.G.); 7 United States Army Research Laboratory, Adelphi, MD 20783, USA; stephen.j.lee28.civ@mail.mil; 8 Navy Medical Center—San Diego, San Diego, CA 92134, USA; huu@g.clemson.edu; 9 School of Kinesiology and Recreation, Illinois State University, Normal, IL 61761, USA

**Keywords:** exercise training, overtraining, sports, strength and conditioning, autonomic nervous system, football players

## Abstract

Current metrics like baseline heart rate (HR) and HR recovery fail in predicting overtraining (OT), a syndrome manifesting from a deteriorating autonomic nervous system (ANS). Preventing OT requires tracking the influence of internal physiological loads induced by exercise training programs on the ANS. Therefore, this study evaluated the predictability of a novel, exercise cardiac load metric on the deterioration of the ANS. Twenty male American football players, with an average age of 21.3 years and body mass indices ranging from 23.7 to 39.2 kg/m^2^ were included in this study. Subjects participated in 40 strength- and power-focused exercise sessions over 8 weeks and wore armband monitors (Warfighter Monitor, Tiger Tech Solutions) equipped with electrocardiography capabilities. Exercise cardiac load was the product of average training HR and duration. Baseline HR, HR variability (HRV), average HR, and peak HR were also measured. HR recovery was measured on the following day. HRV indices assessed included the standard deviation of NN intervals (SDNN) and root mean square of successive RR interval differences (rMSSD) Linear regression models assessed the relationships between each cardiac metric and HR recovery, with statistical significance set at α < 0.05. Subjects were predominantly non-Hispanic black (70%) and aged 21.3 (±1.4) years. Adjusted models showed that exercise cardiac load elicited the strongest negative association with HR recovery for previous day (*β* = −0.18 ± 0.03; *p* < 0.0000), one-week (*β* = −0.20 ± 0.03; *p* < 0.0000) and two-week (*β* = −0.26 ± 0.03; *p* < 0.0000) training periods compared to average HR (βetas: −0.09 to −0.02; *p* < 0.0000) and peak HR (βetas: −0.13 to −0.23; *p* < 0.0000). Statistically significant relationships were also found for baseline HR (*p* < 0.0000), SDNN (*p* < 0.0000) and rMSSD (*p* < 0.0000). Exercise cardiac load appears to best predict ANS deterioration across one- to two-week training periods, showing a capability for tracking an athlete’s physiological tolerance and ANS response. Importantly, this information may increase the effectiveness of exercise training programs, enhance performance, and prevent OT.

## 1. Introduction

Overtraining (OT) manifests from a deteriorating autonomic nervous system (ANS) due to an imbalance between training load and recovery [1]. Among its many functions, the ANS regulates the activity of the cardiac system in response to changes in physiological stimuli (e.g., O_2_ demand during exercise) [2,3]. Thus, any deficiencies in the ANS may impair cardiac function, subsequently reducing exercise capacity and sports performance [4]. The absence of observable, external warning signs specific to OT presents significant challenges. Upon reaching OT, an athlete requires an extensive period of rest for full recovery [5]. Therefore, identifying metrics that accurately assess the physiological tolerance of athletes is critical for optimizing exercise training, enhancing performance, and avoiding OT.

Currently, the measures of cardiac function like baseline heart rate (HR) and heart rate variability (HRV) are used as reliable indicators of OT, as athletes often exhibit abnormal values when in OT [5,6]. A significant limitation of these metrics is their inability to predict early ANS deterioration, leaving athletes and coaches no opportunities for avoiding OT. Moreover, a large proportion of studies previously narrowed their focus to evaluating HR recovery, a metric representing the ANS response, to a single bout of high intense exercise training [7]. HR recovery responses were typically monitored in the acute period up to 72 h post-exercise [8]. OT, however, occurs consequent to repeated bouts of high intensity exercise training coupled with inadequate recovery [1]. Thus, these studies provided limited information about tracking the ANS response to chronic high intensity exercise training and the potential prevention of OT. Another significant limitation of current research is the absence of metrics accurately quantifying the physiological load endured by cardiac muscles during exercise training. Current metrics merely quantify the intensity of an exercise training session, providing an incomplete estimation of the total physiological load [9]. Additionally, determining the level of intensity relies on using maximum HR and HR-reserve. These methods are highly variable and falsely imply a universal maximum HR of 220 beats per min and equivalent age-related declines in cardiac function across all populations [10]. Consequently, these measures likely provide inaccurate, indirect estimates of the physiological load.

Lastly, an increasing number of studies use HRV metrics. HRV is a systemic metric that constantly measures the interplay between the parasympathetic and sympathetic nervous systems [11]. HRV, defined as the time variation between each heartbeat, is sensitive to many non-specific changes in physiological stimuli including respiration, hormonal reactions, metabolic processes, stress, and recovery [11] Thus, fluctuations in HRV are difficult to discern, leading to inconclusive evidence on the direction and magnitude of its response and adaptation to exercise training [12]. These significant limitations highlight the need for a metric that accurately measures the physiological load on the cardiac muscles and physiological tolerance of each athlete. With this metric, coaches may be able to monitor the physiological impact of short- and long-term exposures to high intensity exercise training and determine the appropriate amount of recovery time. This information may lead to more effectively designed exercise training programs, specific to each athlete, enhancing their performance and preventing OT. 

Therefore, the purpose of this study was to evaluate a novel metric that directly quantified the physiological load placed on the cardiac muscle (“exercise cardiac load” herein) during daily and weekly strength- and power-focused exercise training in Division I collegiate football athletes. Existing exercise cardiac metrics including baseline HR, average HR, peak HR, and select HRV indices (SDNN and rMSSD) were analyzed for comparative purposes. We hypothesized that the exercise cardiac load quantified for both daily and weekly training sessions would better predict ANS deterioration than existing cardiac metrics. Specifically, we anticipated that exercise cardiac load would exhibit a strong, positive association with baseline HR and HR recovery 24 h post-exercise training, reflecting reduced ANS recovery and function, respectively. Additionally, we hypothesized a stronger association for cumulative exposures to high exercise cardiac loads compared to acute exposures.

## 2. Materials and Methods

### 2.1. Study Design

This study employed a prospective study design among sample of Division I collegiate male American football players. All cardiac measures including exercise cardiac load, average training heart rate, average peak training HR, baseline HR, HRV, and specifically SDNN and rMSSD were measured on all study subjects throughout the 8-week summer football training program. The training cardiac metrics represented the physiological load placed on the cardiac muscles during “active” training. Baseline HR and HRV metrics presented the 24 h *recovery* of the ANS. HR recovery reflected the *function* of the ANS 24 h post training.

### 2.2. Subjects

Subjects were recruited from a Division I collegiate football team located in the southeastern state of Florida, United States. The athletes were participating in an 8-week, summer football training program. The prospective participants were recruited from a pre-selected group of athletes the coaches identified as “starters”, which were athletes that competed in nearly every regulation game and for most of its duration. Importantly, no exclusion criteria for study participation were imposed. The athletes were, on average, 21.3 years of age, classified as obese with body mass indices ranging from 23.7 to 39.2 kg/m^2^. The sample was predominantly non-Hispanic black. Prior to any measurements, the athletes were informed of the benefits and risks of the study and conflicts of interests of all the authors. All athletes participating voluntarily consented to the study. All study protocols followed the ethical principles defined in the declaration of Helsinki and were approved by the university’s Institutional Review Board (IRB #20191223). 

### 2.3. Methodology

#### 2.3.1. Summer Football Training Program

The summer training program ran from the beginning of May to the end of June 2022. This program lasted 9 total weeks with two, 4-week training blocks separated by 1 week of rest. All exercise sessions occurred in the morning between 0600 and 0900. Athletes completed 40 total sessions: 5 consecutive sessions per week. The duration of the sessions averaged 163.5 (±30.8) min and ranged from 90.0 to 240.9 min (~1.5 to 4.0 h). The training load varied daily and between each athlete. All athletes, regardless of position, were exposed to the same strength and power-focused resistance training, speed training (i.e., short-distance sprints), and agility training regimens. Given the prospective nature of this study, no changes in the training programs were made.

#### 2.3.2. Cardiac Measurement 

Participants were fitted with armband monitors equipped with temperature, electrocardiography (ECG), photoplethysmography (PPG), and inertial measurement unit (IMU) capabilities (Warfighter Monitor^TM^ (WFM), Tiger Tech Solutions Inc., Miami, FL, USA). The WFM armbands were previously validated in several diverse subpopulations [13]. Monitors were placed on the posterior aspect of the left upper arm, secured with an elastic band, and worn at the start and throughout each training session. Although the WFM device collected several biometric parameters, only HR and HRV-related variables were analyzed.

#### 2.3.3. Physiological Load Metrics of All Training Sessions

The physiological load of each training session was estimated using several cardiac metrics including exercise cardiac load, average HR, and peak HR.

##### Exercise Cardiac Load

Exercise cardiac load quantified the physiological load endured by the cardiac muscle while “actively training”. An “active training” state was defined as a sustained HR ≥ 85 beats per min (bpm). Thus, exercise cardiac load was the product of the athlete’s average HR and duration (min) of each session and was calculated as follows:

$$Exercise\;Cardiac\;Load\left(total\;heartbeats\right)=Average\;HR\left(bpm\right)*Session\;Duration\left(min\right)$$

The exercise cardiac load was normalized with the largest exercise cardiac load measured from any athlete during the 8-week training program and multiplied by 100. 

##### Average and Peak HR during Training

*Average training HR* was calculated by averaging all the HR values measuring above 85 bpm collected during each training session. Periods where HR values ≤ 85 bpm were defined as “non-active” and represented periods when athletes were not actively training. *Peak training HR* was defined as the highest HR value achieved during each training session.

#### 2.3.4. The Measures of 24 h ANS Recovery and Function

Several cardiac metrics that measured 24 h post training were used as indicators of ANS recovery and function including baseline HR, HRV indices, and HR recovery. Baseline HR and HR recovery are considered the “gold standard” measure of ANS recovery and response, respectively. HRV is shown to correlate well with baseline HR and HR recovery.

##### 24 h Baseline HR

A 24 h baseline HR represented ANS recovery. Baseline HR was measured in the early morning and followed at least four min of inactivity, per established protocols [14]. Specifically, baseline HR was measured prior to the start (0600–0700) of the following day’s exercise training session. Each athlete was required to remain nearly motionless in a seated position for a period of 5 min to collect a “resting” baseline HR.

##### 24 h Heart Rate Variability

HRV is defined as the time variation between heartbeats [15]. The metrics used to evaluate HRV included the standard deviation of NN intervals (SDNN) and the root mean square of successive differences (rMSSD), described in detail elsewhere [16]. These metrics were calculated during a 5 min interval where the athletes were seated nearly motionless prior to the start of each training session. 

##### 24 h HR Recovery

HR recovery was measured during the next-day’s exercise training session to track ANS function following acute bouts of exercise. HR recovery was defined as the reduction in HR during 30 s rest intervals representing localized parasympathetic activation. HR recovery was measured within the first 30 s of rest as, during this period, HR exhibits the greatest rate of change [17]. HR recovery was quantified for all rest intervals occurring throughout the training session and then averaged. 

**Figure 1:**
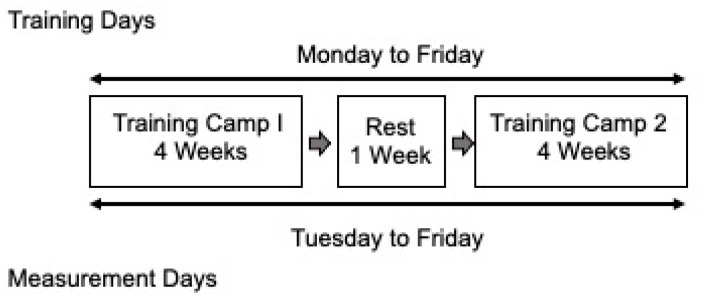
A Schematic of the 8-Week Summer Football Training Camp.

Importantly, baseline HR and HR recovery were measured 24 h following a training session. As such, baseline HR and HR recovery were not measured following one or more rest days. Including rest days would likely dilute the association and not accurately represent the acute and chronic influence of the physiological training load on ANS recovery and function (see Figure 1). 

### 2.4. Statistical Analyses

This study sought to understand the associations between the cardiac metrics of daily and weekly training sessions and the ANS recovery and function. For daily sessions, the cardiac metrics, representing physiological load, were averaged across the 8 training weeks. Exercise cardiac load, average training HR, and peak training HR served as the independent variables. For weekly sessions, one- and two-week averages of exercise cardiac load, average training HR and peak HR served as the independent variables. The one- and two-week averages represented the physiological loads of the previous 5 and 10 training sessions, respectively. Similar calculations were performed for baseline HR, SDNN, and rMSSD, and these metrics also served as independent variables. Next-day HR recovery served as the primary outcome variable. Associations were quantified using two-tailed, linear regression models and were performed separately for each metric. For all models, *β* coefficients and standard errors were estimated, and the a priori threshold for statistical significance was set at α = 0.05. Statistical analyses were performed in MATLAB, version 2021b (MathWorks, Natick, MA, USA).

## 3. Results

Table 1 displays the cardiac and ANS recovery of the athletes during the 8-week summer training program. The average number and duration of the sessions completed were 40 and 163.5 (±30.6) min, respectfully. The athletes, on average, elicited a baseline HR of 62.6 (±6.9) bpm, ranging between 46.3 and 80.5 bpm. During the conditioning sessions, athletes exhibited an average HR of 133.3 (±8.4) bpm, ranging between 111.4 and 164.1 bpm and a peak HR of 167.1 (±9.7) bpm, ranging between 140.3 and 194.4 bpm. The average exercise cardiac load to which the athletes were exposed was 19,776.6 (±3837.8) heartbeats, ranging between 10,016.1 and 30,507.8 heartbeats per session. HR recovery following the exercise cardiac load of the previous conditioning session was, on average, 27.7 (±6.2) bpm, ranging between 11.2 and 47.4 bpm. Lastly, the SDNN and rMSSD indices of athlete HRV were on average, 80.5 (±18.9) milliseconds, ranging between 40.0 and 119.9 milliseconds; and 62.6 (±17.3) milliseconds, ranging between 18.0 and 102.2 milliseconds, respectively.

**Figure 2:**
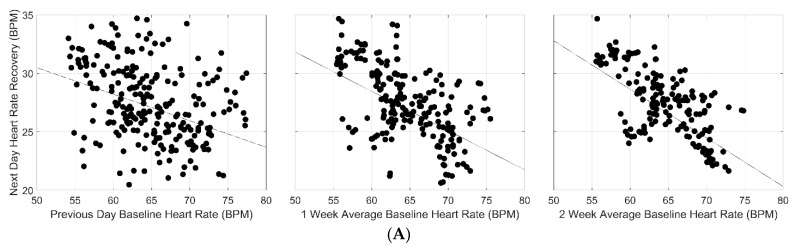

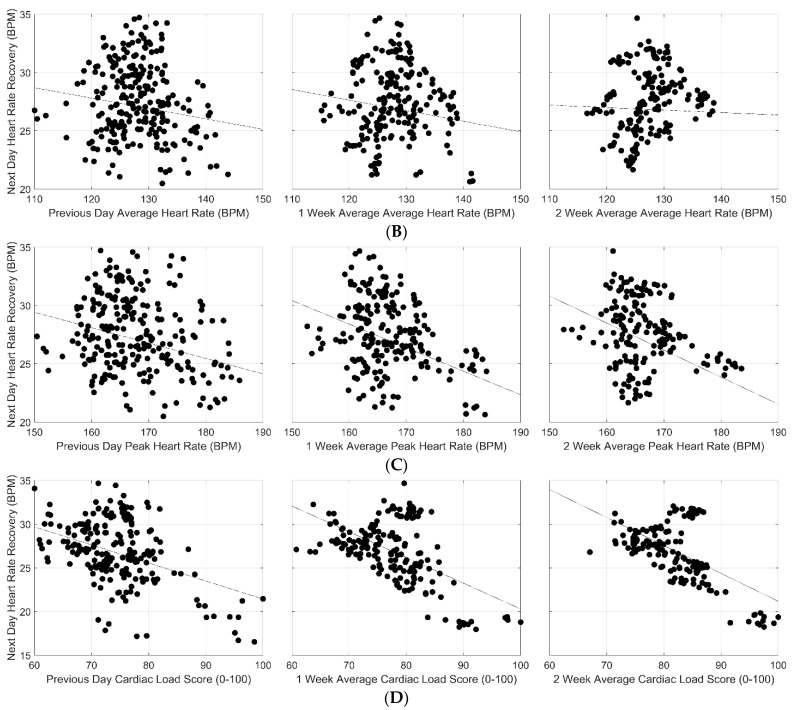
(**A**) Linear Associations Between Previous Day, 1- and 2-Week Baseline HR, and Next-Day HR Recovery. (**B**) Linear Associations Between Previous Day, 1- and 2-Week Average HR, and Next-Day HR Recovery. (**C**) Linear Associations Between Previous Day, 1- and 2-Week Peak HR, and Next-Day HR Recovery. (**D**) Linear Associations Between Previous Day, 1- and 2-Week Exercise Cardiac Load, and Next-Day HR Recovery.

Adjusted linear regression and correlation coefficients representing the associations between several cardiac metrics and next-day HR recovery are presented in Table 2. For baseline HR, a statistically significant negative association with next-day recovery was observed with an increasing magnitude (*β* range: −0.42 to −0.23; *p* < 0.0000) in the slope of this relationship for both daily and weekly exposures to exercise training. Statistically significant negative associations were also observed for average HR (*β* range: −0.09 to −0.02; *p* < 0.0000) and peak HR (*β* range: −0.23 to −0.13; *p* < 0.0000) and next-day HR recovery, albeit lower in magnitude compared to baseline HR. These associations were shown across both daily and weekly exposures to training sessions with a progressive increase in magnitude of the slope observed only for peak HR. Interestingly, exercise cardiac load (total heart beats occurring during a single training session) exhibited the strongest, statistically significant negative association with next-day HR recovery following a 2-week exposure to training sessions, with longer exposures resulting in greater decreases in next-day HR recovery. Like peak HR, the magnitude of the relationship between exercise cardiac load and next-day HR recovery progressively increased across both daily and weekly exposures to exercise training (*β* range: −0.26 to −0.28; *p* < 0.0000). Graphical representations of these relationships appear in Figure 2A–D.

**Figure 3:**
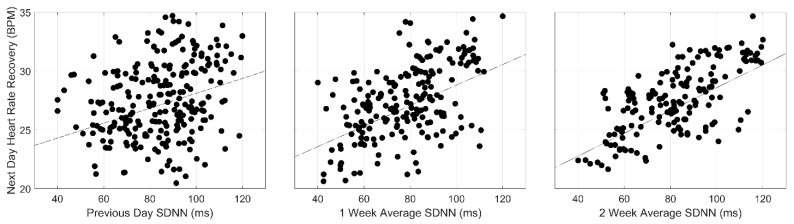
Linear Associations Between Previous Day, 1- and 2-Week Heart Rate Variability, and Next-Day HR Recovery. rMSSD (top) and SDNN (bottom).

The associations between HRV, represented by SDNN and rMSSD indices, and next-day HR recovery are also shown in Table 2. Statistically significant positive associations between both the indices of HRV and next-day HR recovery were observed. Additionally, increasing magnitudes in the slopes were observed across both daily and weekly exposures to training (*β* = 0.06, 0.09, 0.10 and 0.04, 0.07, 0.09; *p* < 0.0000, respectively). Interestingly, compared to baseline HR, peak HR, and cardiac load, the magnitudes of the slopes for SDNN and rMSSD were smaller and in opposing directions. Graphical representations of these associations are displayed in Figure 3.

## 4. Discussion

The purpose of this study was to evaluate the associations between daily and weekly exposures to high intensity, training sessions and the response of the ANS in a sample of Division I football athletes. The major findings of this study were (1) the exercise cardiac load metric exhibited stronger, negative relationships with next-day HR recovery compared to average and peak training HRs, (2) progressive increases in the relationships for exercise cardiac load and peak HR were observed across both daily and weekly exposures to training sessions, and (3) positive associations were observed for HRV metrics; although statistically significant, the strengths of the relationships for SDNN and rMSSD were smaller in comparison to all cardiac metrics.

A novel aspect of this study was that the exercise cardiac load metric introduced in this study exhibited stronger relationships with next-day HR recovery than the other cardiac training metrics. This finding suggests that for high intensity training sessions, exercise cardiac load best predicts ANS deterioration. The exercise cardiac load metric differs considerably from other cardiac training metrics used in this study and others [18,19]. Exercise cardiac load measures the total number of heartbeats occurring in an “active state”, directly quantifying the physiological load endured by the cardiac muscle. Conversely, other metrics like average training HR, peak training HR, HR reserve, etc., simply quantify exercise intensity at a glimpse, which identifies the level of effort at which an athlete is actively working [9,20]. Consequently, these metrics only partially quantify the physiological load endured by the cardiac muscle during exercise training [21]. Moreover, exercise intensity, usually expressed as a percentage of cardiac capacity (e.g., %HR maximum, % peak HR, %HR reserve) is calculated using flawed equations and assumptions. For example, without consistent empirical support, these equations assume that all individuals elicit a 220-bpm maximum cardiac rate that linearly declines with age and that resting HR is accurately approximated in a non-rested state [10]. These significant limitations likely explain the lower magnitudes observed in this study for the average HR and peak HR associations with next-day HR recovery. Interestingly, in this study, exercise cardiac load elicited a lower magnitude of the association with next-day HR recovery compared to baseline HR. Importantly, this observation does not suggest that baseline HR is a better metric for predicting ANS deterioration. Unlike exercise cardiac load, baseline HR is primarily used for determining, at a given point in time, whether an athlete reached an OT state. As such, baseline HR is not capable of predicting ANS deterioration but rather serves as a useful criterion for diagnosing OT [20]. Taken together, the exercise cardiac load directly assesses the physiological load induced on cardiac muscles, potentially providing the accurate tracking of each athlete’s physiological tolerance and predictions of ANS deterioration and OT.

Another unique finding of this study was the observation of progressively increasing strength of the relationships between exercise cardiac load and next-day HR recovery across longer-term exposures to high intensity training sessions. This finding supports the existing literature that consistently shows that ANS deterioration occurs consequent to repeated exposures of high intensity exercise training followed by inadequate recovery [22]. In this study, the football athletes participated in 5 consecutive days of high intensity sessions of considerably long duration (90.0 to 240.9 min). At the end of each week, athletes were given a 48 h recovery period. Interestingly, in additional analyses (data not shown), the relationship between exercise cardiac load and next-day HR recovery weakened when comparing the HR recovery on Monday of the following week to the cardiac load of the previous Friday’s session. This observation might suggest that a 48 h period allows for, in this sample of athletes, sufficient recovery time. However, the increased strength of the negative association between exercise cardiac load and next-day HR recovery from the one-week to two-week cumulative exposure contradicts this notion. In fact, the latter observation highlights the exacerbated ANS deterioration consequent to insufficient recovery. Moreover, this observation emphasizes the utility of tracking the physiological load endured by the cardiac muscle and the response of the ANS. For coaches, this information may identify the athlete’s physiological tolerance, subsequently indicating requisite modifications to their training program to potentially avert further ANS deterioration and prevent OT. 

Notably, this study observed statistically significant, positive associations between HRV indices and next-day HR recovery. This finding suggests that increases in HRV indices following daily and/or weekly exposures to high intense training loads may indicate a sufficient recovery of the ANS. While this finding is supported by some scientific studies, others refute the ability of HRV indices to accurately reflect the ANS response [6,12]. In support, studies previously showed that increases in HRV indices were positively associated with ANS recovery following acute and chronic bouts of endurance exercise training. Conversely, others reported that these same trends led to functional overreaching [23], a state immediately preceding overtraining. Another study demonstrated that declines in HRV, a suggested indicator of ANS deterioration, found among functionally overreaching athletes were associated with improved performance [24]. The inconclusive evidence is likely attributable to the increased complexity of HRV in addition to its high sensitivity to non-specific changes in physiological stimuli. Thus, until a more concrete understanding of the responses and adaptations of HRV to exercise training is reached, its use in tracking and predicting ANS deterioration may be inappropriate. Of interest, compared to the exercise cardiac load metric evaluated in this study, the magnitudes of the associations for SDNN and rMSSD appeared smaller (*β* range: −0.26 to −0.18 vs. 0.04 to 0.09, respectively). This finding may further support the use of the exercise cardiac load metric for tracking and predicting ANS deterioration in athletes.

### Strengths and Limitations

This study possesses a few strengths and weaknesses warranting attention. First and foremost, this study introduced a novel metric that directly assessed the physiological load placed on the cardiac muscles, which was strongly associated with ANS deterioration. As such, the exercise cardiac load metric may provide sport coaches with an accurate and practical tool for identifying each athlete’s physiological tolerance, predicting ANS deterioration, and potentially preventing OT. Second, this study employed a prospective study design in a natural sport setting, likely allowing for a better translation of these findings to similar types of sports. Third, this study assessed the physiological loads of high intensity training, which are scarcely evaluated in the current literature, with a large proportion of studies focusing on endurance exercise training. Given that many contact sports implement training programs, these findings significantly contribute to the scientific literature, as it reaches an understudied area in sports. Fourth, this study evaluated the influence of daily and weekly exposures of high intensity training, providing important information on the longitudinal impact of this type of training on ANS deterioration. The current study is not without its limitations. First, the study sample only included 20 university-aged, adult males competing on a singular football team, potentially reducing the generalizability of the findings. Second, this study did not include female athletes, further restricting the generalizability of this study. Lastly, extraneous factors potentially affecting the ANS including nutritional status and sleep were not measured. 

## 5. Conclusions

Collectively, the observations of this study demonstrated several concepts regarding the physiological load of exercise and the response of the ANS, specifically for sports implementing training programs. First, our study introduces a novel metric that strongly predicts the potential deterioration of the ANS induced by exercise training and outperforms existing cardiac metrics like baseline HR and HR recovery. Second, repeated exposures to high intensity training with minimal recovery exacerbates the deterioration of ANS, highlighting the need for a longitudinal tracking of the cardiac loads in exercise training programs. Additionally, our study suggests a potential misuse of HRV consequent to its increased complexity and sensitive nature. For future studies aiming to further understand the influence of exercise training on the response of the ANS, the use of exercise cardiac load as described in this study or similarly designed metrics in addition to including several longitudinal timepoints are strongly encouraged. Moreover, future studies should include samples of female athletes and athletes of similar sports.

## 6. Practical Implications

The ECL metric is a novel, practical, and simple measure of an athlete’s physiological tolerance to exercise training. This metric allows coaches to track the influence of acute and cumulative exercise training on the ANS of each athlete to (1) prevent declines in sport performance, functional overreaching, and overtraining; (2) individualize programs that train athletes within their physiological reserve; and (3) optimize training programs and sport performance.

## Figures and Tables

**Table 1:** Cardiac Metrics and ANS Recovery During a Summer 8-Week Football Training Program in Division I Collegiate Athletes.

	Summer Football Training Program					
	8 Weeks	1st Week	4th Week	8th Week	1st, 4-Week Block	2nd, 4-Week Block
**Cardiac Metrics**						
*Average HR (bpm)*	133.3 (8.4)	132.8 (5.7)	133.6 (11.1)	129.9 (6.9)	134.6 (8.8)	132.0 (7.6)
*Peak HR (bpm)*	167.1 (9.7)	167.3 (7.3)	165.4 (11.0)	164.9 (9.8)	167.6 (9.6)	166.6 (9.7)
*Cardiac Load* *(total heart beats)*	19,776.6 (3837.8)	19,358.7 (2840.9)	19,322.1 (4372.2)	18,067.5 (3756.2)	19,550.9 (3476.6)	20,008.4 (4170.7)
*SDNN (ms)*	80.5 (18.9)	84.5 (14.2)	72.3 (15.8)	77.5 (17.4)	76.5 (17.4)	84.7 (17.4)
*rMSSD (ms)*	62.6 (17.3)	68.3 (15.5)	51.1 (12.6)	53.3 (15.5)	58.3 (15.5)	64.3 (15.5)
**ANS Recovery**						
***HR Recovery (bpm)***	27.7 (6.2)	28.4 (4.8)	26.0 (6.3)	28.4 (6.6)	27.2 (5.7)	28.2 (6.7)
***Baseline HR (bpm)***	62.6 (6.9)	64.6 (6.6)	62.6 (7.1)	61.3 (5.9)	63.5 (6.7)	61.8 (6.9)

**Table 2:** Adjusted Linear Associations Between Cardiac Metrics and ANS Deterioration in Division I Collegiate Football Athletes.

	Slope (*β*)	SE	Adjusted R^2^	*p*-Value
**Cardiac Metrics**				
**Baseline HR (bpm)**				
*Previous Day*	−0.23	0.04	0.43	<0.0000
*1-Week*	−0.34	0.05	0.55	<0.0000
*2-Week*	−0.42	0.05	0.62	<0.0000
**Average HR (bpm)**				
*Previous Day*	−0.09	0.04	0.23	<0.0000
*1-Week*	−0.09	0.05	0.23	<0.0000
*2-Week*	−0.02	0.06	0.13	<0.0000
**Peak HR (bpm)**				
*Previous Day*	−0.13	0.03	0.35	<0.0000
*1-Week*	−0.20	0.04	0.46	<0.0000
*2-Week*	−0.23	0.04	0.49	<0.0000
**Cardiac Load (total heart beats)**				
*Previous Day*	−0.18	0.03	0.61	<0.0000
*1-Week*	−0.20	0.03	0.69	<0.0000
*2-Week*	−0.26	0.03	0.71	<0.0000
**SDNN (ms)**				
*Previous Day*	0.06	0.01	0.38	<0.0000
*1-Week*	0.09	0.01	0.51	<0.0000
*2-Week*	0.09	0.01	0.61	<0.0000
**rMSSD (ms)**				
*Previous Day*	0.04	0.01	0.30	<0.0000
*1-Week*	0.07	0.01	0.46	<0.0000
*2-Week*	0.09	0.01	0.53	<0.0000

## Data Availability

Data presented in the current paper are available upon request.
